# A conceptualization and psychometric evaluation of positive psychological outcome measures used in adolescents and young adults living with HIV: A mixed scoping and systematic review

**DOI:** 10.1371/journal.pgph.0002255

**Published:** 2024-08-12

**Authors:** Jermaine M. Dambi, Frances M. Cowan, Faith Martin, Sharon Sibanda, Victoria Simms, Nicola Willis, Sarah Bernays, Webster Mavhu

**Affiliations:** 1 Rehabilitation Sciences Unit, Faculty of Medicine and Health Sciences, University of Zimbabwe, Harare, Zimbabwe; 2 Centre for Sexual Health and HIV/AIDS Research (CeSHHAR), Harare, Zimbabwe; 3 Liverpool School of Tropical Medicine, Liverpool, United Kingdom; 4 Cardiff University, Park Place, United Kingdom; 5 London School of Hygiene & Tropical Medicine, London, United Kingdom; 6 ZVANDIRI, Avondale, Harare, Zimbabwe; 7 University of Sydney, Camperdown, Australia; PLOS: Public Library of Science, UNITED STATES OF AMERICA

## Abstract

**Introduction:**

Sub-Saharan Africa bears the greatest burden of HIV, with comorbid mental conditions highly prevalent in people living with HIV. It is important to evaluate the mental health of adolescents and young adults living with HIV (AYALHIV) comprehensively by measuring both negative and positive psychological constructs. There has been a proliferation of interest in positive psychological outcome measures, but the evidence of their psychometric robustness is fragmented. This review sought to: 1) Identify positive psychological outcomes and corresponding outcome measures used in AYALHIV in sub-Saharan Africa. 2) Critically appraise the psychometrics of the identified outcome measures.

**Methods and analysis:**

Two reviewers independently searched articles in PubMed, Scopus, Africa-Wide Information, CINAHL, Psych INFO and Google Scholar. Searches were conducted from November 2022 to February 2023. Two separate reviewers independently reviewed retrieved articles. We applied a narrative synthesis to map the key constructs. The risk of bias across studies was evaluated using the COnsensus-based Standards for the selection of health Measurement INstruments (COSMIN) checklist. The quality of the psychometric properties was rated using the COSMIN checklist and qualitatively synthesized using the modified Grading of Recommendations Assessment, Development, and Evaluation checklist.

**Results:**

We identified 15 positive psychological constructs: body appreciation, confidence, coping, flourishing, meaningfulness, personal control, positive outlook, resilience, self-management, self-compassion, self-concept, self-efficacy, self-esteem, self-worth and transcendence, that had been used to assess ALHIV. The most measured constructs were resilience, self-concept, self-esteem, coping and self-efficacy. Construct validity and internal consistency were the properties most frequently considered, while content validity and structural validity were assessed less often.

**Conclusions:**

Few studies performed complete validations; thus, evidence for psychometric robustness was fragmented. However, this review shows the initial evidence of the feasibility of using positive psychological outcomes in low-resource settings. Instead of creating new outcome measures, researchers are recommended to leverage the existing measures, adapt them for use and, if appropriate, strive to maintain the factorial structure to facilitate comparisons.

**Registration:**

PROSPERO-CRD42022325172.

## Introduction

The burden of HIV in young people in low-resource settings, particularly sub-Saharan Africa (SSA), is disproportionately high [1–3]. According to UNAIDS estimates for 2010–2022, SSA accounted for 51% of global new HIV infections [3]. Adolescence and early adulthood are challenging developmental stages, with the burden of navigating life challenges often greater for adolescents and young adults living with HIV (AYALHIV) [2, 4, 5]. For instance, AYALHIV face HIV-related stigma, which affects their ability to negotiate reproductive health and predisposes them to social isolation and socioeconomic deprivation [6, 7]. Further, AYALHIV are also at a higher risk of grief, orphanhood and other difficulties, compared to their HIV-negative peers [1, 2, 5, 8].

HIV has evolved into a long-term condition with a concurrent surge in comorbid non-communicable diseases [4, 9]. For example, common mental conditions, including anxiety and depression, are highly prevalent in AYALHIV, with a pooled prevalence of 25–48% compared to 14% prevalence in adolescents and young adults in the general population [2, 10]. However, few integrated programs combine HIV and mental health care [2, 4, 5]. Notably, many mental conditions that present in adulthood emerge in late adolescence and young adulthood, and effective management earlier in the life course can prevent long-term mental health difficulties [2, 4]. Systematic reviews have demonstrated that access to mental healthcare by AYALHIV is associated with positive outcomes across the treatment continuum, including: increased treatment initiation, treatment adherence, retention in care, viral suppression, and reduced morbidity and mortality [4, 5, 11, 12].

Mental healthcare endpoints within HIV care have traditionally been conceptualized as negative psychiatric symptomatology improvements [8, 13]. For example, success in psychotherapies is invariably benchmarked against declines in rates of depression, anxiety, post-traumatic stress disorders and other negative psychological indices [5]. However, focusing on negative indices misses the opportunity to capture mental health’s multidimensionality [14]. A holistic mental health evaluation requires a comprehensive focus on both negative and positive mental health constructs [5, 7], and recognition of this has resulted in a shift towards positive psychology, a branch of psychology that emphasizes increasing human well-being and positive functioning [8, 14–16]. Although positive psychology is not universally conceptualized and defined, Park et al (2016) propose four pillars to positive psychology i.e., positive subjective experiences (e.g., happiness, gratification); positive individual traits (e.g., character strengths); positive interpersonal relationships (e.g., friendship, marriage) and positive institutions (e.g., families, communities) [17]. Positive mental health interventions (PMHIs) are anchored upon optimizing human strengths and capabilities to improve positive outcomes such as self-esteem, resilience, hope, self-worth, social resources and flourishing [8, 13, 18]. For instance, studies have shown that people with chronic conditions (e.g., HIV) develop resilience with time [8, 18]. The resilience developed in navigating the challenges of living with a chronic condition is potentially transferable into everyday functioning [18]. Positive psychology interventions (e.g., resilience-building approaches) are central to prevention and health promotion and act as an entry point to stepped care for mental health problems in routine HIV care [8].

With the proliferation of PMHIs comes the need to routinely evaluate the clinical endpoints from both the clients’ and therapists’ perspectives [19]. The patient’s evaluation of their health, treatment expectations and outcomes are contingent upon the availability of validated and reliable outcome measures [13]. The last few decades have seen a proliferation of positive psychology outcome measures [20]. However, there is limited understanding of the salient positive psychological constructs linked to AYALHIV’s improved well-being and health-related quality of life. Rigorous evaluation of PMHIs is essential but limited by a lack of robust measures.

In their scoping review, Wayant et al. (2021) mapped 15 positive psychological constructs associated with increased quality of life and survival in adolescents and young adults living with cancer [21]. Well-being, personal growth, hope, meaning in life, self-esteem, vitality and optimism were the most cited positive constructs [21]; these are potentially relevant to AYALHIV. Conversely, etiological differences between cancer and HIV could also lead to differences in lived experiences, resulting in differential perceptions of positive psychological constructs [21]. For instance, HIV-related stigma (internalized and enacted stigma) may have a more significant impact on mental health functioning in AYALHIV [12, 22] when compared to the effects of cancer-related stigma [13]. It is thus critical to contextualize the impacts of positive psychological outcomes in AYALHIV.

Elsewhere, Govindasamy et al. (2021) performed a mixed-methods systematic review to explore correlates of well-being among AYALHIV in SSA to inform econometric evaluations [22]. The study showed that social support, belonging, purpose in life and self-acceptance optimize well-being in AYALHIV [22]. Also, Orth, Moosajee and Van Wyk (2023) performed a systematic review to identify and conceptualize mental wellness in adolescents [14]. The review identified 13 concepts: life satisfaction, mental well-being, resilience, self-efficacy, self-esteem, connectedness, coping, self-control, mindfulness/spirituality, hope, sense of coherence, happiness and life purpose. However, no psychometric evaluation of the analyzed instruments was done [14, 22]. A critical appraisal of the identified outcome measures is important to provide a repository of outcome measures for use in future studies (observational and interventional study designs) evaluating positive psychological constructs.

Earlier work by Govindasamy et al. (2021) and Orth, Moosajee and Van Wyk (2023) provides essential insights into the broader nature of well-being conceptualization in AYALHIV. However, a lack of nuanced understanding of the measurement properties of the corresponding outcome measures for the constructs identified in the two reviews limits our comprehensive understanding of the spectrum of positive psychological constructs in AYALHIV living in SSA. There is a need to build on earlier work and understand positive psychological constructs in HIV care for AYALHIV: such work is potentially applicable to other chronic conditions, given the multi-level impacts of HIV. Also, there is a lack of collective evidence of the psychometric robustness of the positive psychological outcome measures used in AYALHIV. Some of the available generic outcomes may not comprehensively reflect the nuances of living with HIV [22]. Further, different investigators use varying wording to refer to the same construct; mapping the constructs is vital. This mixed review, therefore, sought to:

1. Identify positive psychological outcomes and corresponding outcome measures used in AYALHIV in SSA.
2. Critically appraise the psychometric properties of the identified positive psychology outcomes used in AYALHIV.

## Methods

### Overview

This mixed review was done in two sequential and complementary phases. First, a scoping review identified positive psychological outcomes in AYALHIV in SSA and used a narrative synthesis to qualitatively map the constructs onto the corresponding measures. The scoping review was performed per Preferred Reporting Items for Systematic Reviews and Meta-Analyses extension for Scoping Reviews (PRISMA-ScR) guidelines—See S1 Table [23]. The second phase systematically evaluated the psychometric properties of the outcomes identified from the scoping review by critically appraising the methodologies and quality of reported measurement properties. Evaluation of outcome measures’ psychometrics was performed and reported according to the Preferred Reporting Items of Systematic Reviews and Meta-Analyses (PRISMA) guidelines [24]—See S2 Table. Where appropriate, we outlined specific methodological considerations unique to each phase.

### Protocol / registration

The protocol was registered on the PROSPERO database—CRD42022325172 and was previously published [16].

### Eligibility criteria

Table 1 shows the study’s eligibility criteria, including the rationale.

**Table 1:** Studies eligibility criteria.

	Inclusion criteria and rationale	Exclusion criteria and rationale
**Population**	▪ Adolescents and young adults living with HIV (AYALHIV). We focused on AYALHIV as this is the group with the greatest burden of HIV globally [22]. ▪ In cases of studies containing AYALHIV and other age bands, studies were only included if the average age was within the 10–24 years range or if >50% of the participants were AYALHIV.	▪ If the average age was outside the 10–24 years range ▪ If <50% of the study participants were AYALHIV.
**Constructs**	▪ Positive psychological constructs broadly defined as any construct focusing on "… aspects of the human condition that promote fulfillment, happiness and flourishing…" [25]. ▪ Positive psychology is a rapidly developing field; consequently, there is variability in the definition and conceptualization of psychological constructs [8, 14–16, 21, 25]. We build upon operational definitions outlined by Wayant et al. in their mapping of positive psychological constructs in pediatric and adolescent/young adult patients with cancer [21]. Wayant et al.’s scoping review yielded these 15 constructs: contentment, gratitude, happiness, hope, life satisfaction, meaning in life, optimism, perseverance, personal growth, resilience, self-esteem, self-acceptance, tranquility, vitality and well-being.	▪ Composite constructs such as quality of life, which may include certain elements of positive psychological constructs.
**Time**	▪ No time restriction	▪ N/A
**Setting**	▪ Sub-Saharan Africa ▪ Any research setting e.g., community, facility-based, amongst other settings.	
**Study designs**	▪ All primary quantitative designs i.e., experimental (e.g., RCTs), observational (e.g., cohort, case-control, cross-sectional) ▪ Mixed methods studies ▪ All qualitative study designs	▪ Reviews (e.g., systematic or scoping reviews) ▪ Editorials ▪ Study protocols
**Language**	English	Non-English languages as we did not have the resources to analyze articles published in other languages.

### Information sources

For the systematic review, we only included peer-reviewed articles, as our focus was on outcome measures with known evidence of psychometric robustness. Peer-reviewed articles were searched, filtered by peer review status and retrieved from these electronic databases: PubMed, Scopus, Web of Science, Africa-Wide Information, CINAHL, PsychInfo, and Google Scholar. Databases were searched from inception through February 28^th^, 2023; there was no restriction by publication date to ensure literature/information saturation. Where only an abstract was available online, and information regarding psychometrics was neither clear nor available from the text, an attempt to contact the lead author was made, requesting the full article to ensure literature saturation and a truthful rating. The article was excluded from the review if there was no response in two weeks following three email reminders. The scoping review broadly aimed to identify the most used positive psychological constructs in AYALHIV in SSA; this necessitated the inclusion of non-peer-reviewed information sources. In addition to the peer-reviewed articles, we also reviewed grey literature using the Google Scholar search engine to search potential databases such as university databases, research reports, pre-prints, newsletters and bulletins, policy briefs, guidelines and conference proceedings for articles. We also performed backward and forward searches of the reference lists of identified articles and databases for completeness. Finally, we contacted experts implementing PMHIs to check for articles we missed during the literature searches.

### Search strategy

For the scoping review, as an illustration, articles in CINAHL were searched using the AND Boolean logic operators, i.e., 1 AND 6 AND 9 AND 12 (S3 Table). The search strategy for the systematic review component was amended to include additional constructs identified through the scoping review.

### Data management

Retrieved articles were imported into a password protected Mendeley reference manager. The articles were also synchronized onto Mendeley and Dropbox cloud storage platforms and backed onto a password-encrypted external hard drive. All collaborators had full access/administrative privileges to the shared Dropbox folder for the present systematic review. A trail/history of the electronic searches was also saved on users’ PubMed, Scopus and EBSCOhost accounts. We also printed summaries of all the searches to enhance data capturing of the search records.

### Data collection process

The data collection process was conducted in three stages, i.e., article retrieval, screening and data extraction. These processes were invariably similar for the scoping and systematic review phases. Here, we describe these processes and highlight, where appropriate, differences in the two review phases.

#### Article retrieving

Two researchers (SS & JMD) independently searched articles using a pre-defined search strategy, with no restriction on publication date. The lead author (JMD) then imported the searches into Mendeley and removed duplicates.

#### Screening

Upon completion of article retrieval, senior researchers (SB & WM) and AYALHIV with previous experience in systematic reviews independently screened the articles by title and abstract using Rayyan software [26]. To increase methodological rigor, researchers independently reviewed all retrieved articles, including documenting reasons for exclusion. Rayyan software automatically collates the number of hits assigned different ratings by reviewers. Discrepancies were resolved through discussion, and where consensus was not reached, a more senior researcher (WM) made the final decision. JMD and SS then performed backward and forward citation searches to identify other potential articles. Two senior researchers (FMC & WM) reviewed the list of identified articles afterward to check for the completeness of the searches.

#### Data extraction

Once searches were finalized, two researchers (FM and NW) retrieved the full articles and independently extracted data from articles meeting the inclusion criteria. Disagreements during data extraction were resolved through consensus, and more senior researchers (FMC & WM) made the final decisions if any impasses occurred. For both phases of the review, we extracted the following information per study: research setting and design, study sample and participants’ demographics. For the systematic review component, we extracted information on the mode of administration, the number of items, descriptions of domains, scoring and interpretation of scores, and whether measures were free to use or required a license fee or other payment.

### Charting/outcomes and prioritization

Qualitative conceptualization of positive psychological constructs and the appraisal of psychometric properties of the identified outcome measures were the primary outcomes of the scoping and systematic review phases, respectively. For the systematic review, the clinical utility of the identified outcome measures was the secondary outcome. See S4 Table for operational definitions of psychometric properties for the systematic review component [27, 28].

### Risk of bias-individual studies

The scoping review aimed to understand the conceptualization of AYALHIV’s positive psychological constructs. Consequently, we performed no risk of bias (RoB) assessments. However, the systematic review component aimed to synthesize the evidence of psychometric robustness, necessitating RoB assessment. We used the revised COnsensus-based Standards for the selection of health Measurement INstruments (COSMIN) checklist to assess the RoB across studies retrieved for psychometric evaluation [27, 28]. The COSMIN methodology consists of three steps. The checklist includes methodological benchmarks for ten psychometric properties, which are categorized into three major groups, i.e., content validity (e.g., patient-reported outcome measure development), internal structure (e.g., structural validity) and other psychometrical properties (e.g., criterion validity) [27, 28]. Each psychometric property is rated using a pre-set criterion, and using the principle of "worse score counts", the lowest rating is ascribed as the overall methodological quality rating [28]. Methodological quality is rated on a four-point Likert scale, i.e., "inadequate", "doubtful", "adequate" and "very good"; the higher the rating, the lower the risk of bias [27, 28]. We anticipated that not all details might be recorded for the retrieved articles, especially for studies whose primary aim was not psychometric evaluation. We, therefore, contacted the corresponding author to achieve the most truthful rating of the psychometric property to minimize bias during analysis.

### Quality of psychometric properties and data extraction

The quality of psychometric properties was evaluated using an updated, hybrid checklist based on previous work by Terwee et al. [29] and Prinsen et al. [30] (See S5 Table). Each psychometric property was rated as; sufficient (+), insufficient (–), or indeterminate (?) [27]. Positive ratings represent high-quality psychometrics [27]. The critical appraisal was independently done by two researchers (WM and JMD), with differences resolved through discussion.

### Best evidence synthesis

We applied a narrative synthesis to map the key "themes/constructs" emerging from the scoping review. We mapped the constructs to corresponding outcome measures; this mapping exercise subsequently guided the psychometric evaluation. The collective evidence per psychometric property per outcome was synthesized using the modified Grading of Recommendations Assessment, Development and Evaluation (GRADE) checklist [31]. The modified GRADE checklist was then used to collate the RoB results and the quality of psychometric ratings to qualitatively synthesize/summarize the quality of evidence per psychometric property across studies. A meta-analysis was not possible given the heterogeneity of outcome measures retrieved. The quality of evidence per psychometric property was classified as very low, low, moderate or high [31]—See S6 Table.

### Patient and public involvement statement

We worked collaboratively with AYALHIV during data collection and dissemination. The AYALHIV had variable experiences and competences. For instance, undergraduate AYALHIV who were previously trained and involved in systematic reviews assisted with article screening. This review is attached to ongoing work in which AYALHIV are collaboratively engaged. It is part of a larger study to explore various constructs to understand how they improve AYALHIV’s health outcomes. We have recruited AYALHIV to serve as a Youth Expert Panel (YEP). The YEP functions as both a guide to the study/research process and an additional group of analysts and discussants to examine the emerging analysis and findings. We also co-created the dissemination plans; for instance, adolescents and young adults with lived experiences were involved in co-developing output animation and contributing to the project blogs, amongst other dissemination activities.

### Ethics and dissemination

No ethical approvals were needed as this is a literature review. The mixed review maps and appraises the collective evidence of the psychometric robustness of positive psychological outcomes used in AYALHIV. The review builds on recommendations of systematic reviews on objectively measuring positive psychological constructs across diverse populations. This is important given the need to use valid and reliable outcomes in understanding the positive effects of living with HIV. The review also assisted in identifying psychometrically robust outcomes to inform an item bank to adapt a context-specific outcome measure for AYALHIV in low-resource settings. For example, we consolidated all self-esteem outcome measures and categorized items from multiple outcomes into common factors/"themes". The outputs collectively informed the development, implementation and evaluation of a bespoke positive mental health intervention for AYALHIV; hence, a multimodal dissemination plan is needed to reach multiple stakeholders. In addition to publishing the outcomes in a peer-reviewed journal, we disseminated the outcomes through social media, policy briefs and blogs.

## Results

The results are presented in two parts. First, we present the mapping of constructs identified from the analyzed studies from the scoping review component. The second part presents the qualitative synthesis of standardized outcome measures analyzed in the systematic review.

### Study selection

**Fig 1:**
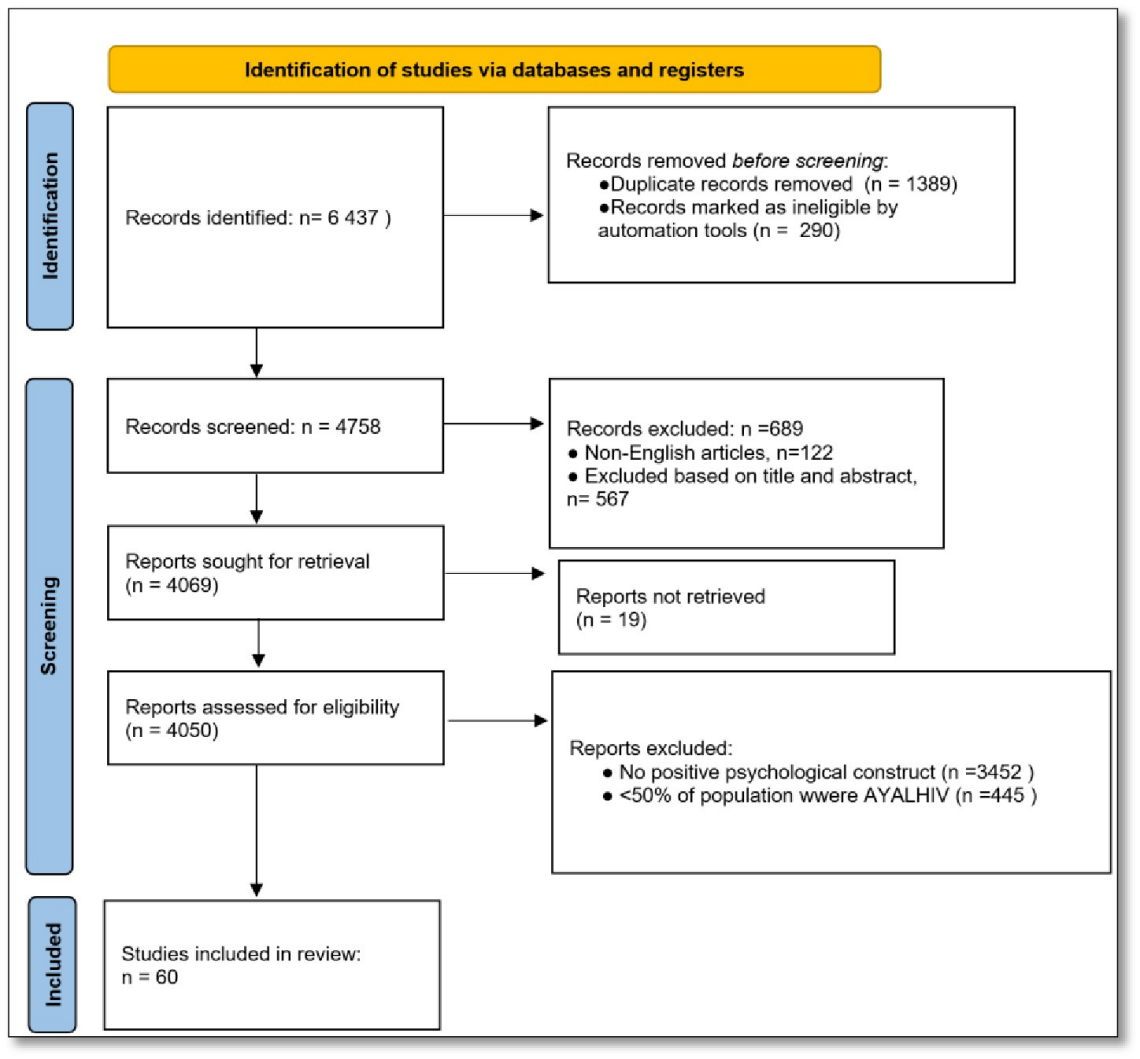
PRISMA study selection flowchart.

We retrieved 6437 studies, of which 1679 were duplicates. After de-duplication, 4748 articles were screened by title and abstract; 4050 were assessed for eligibility. Sixty articles met the full criteria and were analyzed in this review—See Fig 1.

### Description of study participants and settings

Slightly over half of the studies (15/29) used outcome measures that were developed in high-income settings. Of the 29 studies, 19 (66%) were conducted in urban areas, six (21%) in rural settings and four (14%) in both urban and rural localities. 13/29 (45%) studies were cross-sectional, with 20/29 (69%) published between 2018 and 2023 –See Table 2.

**Table 2:** Description of study participants and settings.

Construct	Name of tool	Country; setting	Design	Participants; Sample size	Age (years)
Body Appreciation	Body Appreciation Scale 2 (BAS-2)	South Africa: Urban primary care	Cross-sectional	YPLHIV, (N = 76)	Range:15–24; Mean (SD): 19.4 (2.6)
Confidence	Ad hoc	Zimbabwe: Rural primary healthcare clinics	RCT	ALHIV, (N = 94)	Range: 10–15
Coping	Psychological Adjustment to Illness Scale Self Report (PAIS-SR)	Nigeria; Urban	Quasi-experimental	Non-Disclosed YPLHIV, (N = 19)	Range: 15–29
Coping	Acceptance of Illness Scale (AIS)	Nigeria: Rural and Urban HIV Treatment Centers	Mixed methods	Pregnant women living with HIV, (N = 840)	Range: 22–46
Coping	Ad hoc	South Africa: Primary care clinics	RCT	Women living with HIV, (N = 143)	Range: 18–49
Flourishing	Flourishing Well Being Scale (FWBS)	South Africa; Households	Cross-sectional	Adolescent Girls & Young Women Living with HIV, (N = 568)	Range: 10–24
Meaningfulness	HIV Meaningfulness Scale (HIVMS)	Nigeria: Rural and Urban HIV Treatment Centers	Mixed methods	Pregnant women living with HIV, (N = 840)	Range: 22–46 years
Personal Control	Mastery Scale (MS)	Ghana; Urban hospital-based clinic	Cross-sectional	PLHIV in Ghana & USA, (N = 55 Ghana)	Range: 15–49
Positive Outlook	Positive Outlook-Individual Protective Factors Index (PIPFI)	Uganda: Urban community clinic	Retrospective cohort study	Children living with HIV, (N = 165)	Range: 6–18: Mean (SD): 10.8 (3.5)
Resilience	Child Youth Resilience Measure-12 (CYRM-12)	South Africa; Urban public health ART clinics	Cross-sectional	ALHIV, (N = 385)	Range: 13–18: Median (IQR): 15 (14–16)
Resilience	Connor-Davidson Resilience Scale (CDRS-25)	South Africa: Rural community-based	Cross-sectional survey	YPLHIV, (N = 334)	Range: 12–24: Median (IQR): 21 (16 to 23)
Resilience	Connor-Davidson Resilience Scale (CDRS-25)	South Africa: Rural public healthcare facilities	Cross-sectional survey	YPLHIV, (N = 359)	Range: 12–24: Median (IQR): 21 (16–23)
Resilience	Connor-Davidson Resilience scale (CDRS-10)	South Africa: Households	Cross-sectional	Adolescent Girls & Young Women Living with HIV, (N = 568)	Range: 10–24
Self-Management	Adolescent HIV Self-Management Scale (AdHIVSM)	Lesotho; Urban hospital and Youth center	Cross-sectional survey	AYLHIV, (N = 183)	Range: 15–25: Median (IQR): 22 (4)
Self-Management	Adolescent HIV Self-Management Scale (AdHIVSM)	South Africa: Urban healthcare facilities	Cross-sectional	ALHIV, (N = 385)	Range: 13–18 Median (IQR): 15 (14–16)
Self-compassion	Self-compassion scale (SCS)	Nigeria: Rural and Urban HIV Treatment Centers	Mixed methods	Pregnant women living with HIV, (N = 840)	Range: 22–40
Self-Concept	Beck Youth Self-Concept Scale (BYSCS)	South Africa: Urban HIV clinics	Cohort	YLPHIV, (N = 203), HIV-U (N = 44)	Range: 9–11: Median (IQR) YLPHIV: 10,7 (9.9–11.4) Median (IQR): HIV-U: 10.3 (9.7–11.1)
Self-Concept	Beck Youth Self-Concept Scale (BYSCS)	South Africa: Urban Public Sector Healthcare Service	Prospective cohort study baseline data	HIV+ adolescents, (N = 204), Control (N = 44)	Range: 9–11: HIV+ mean = 10.4 SD 0.9; controls Mean = 10.4 SD = 1.1
Self-Concept	Beck Youth Self-Concept Scale (BYSCS)	South Africa; Urban Research Centre	Cohort study	ALHIV, (N = 122)	Range: 12–15 PHIV+ (Mean SD): 13.5 (1.0): Controls (Mean SD):13.8 (1.2)
Self-Concept	Tennessee Self-concept Scale-2 (TSCS-2)	Uganda; Rural public schools	Longitudinal study	AIDS orphaned adolescents, (N = 268)	Range: 11–17: Mean (SD): 13.7 (1.3)
Self-Concept	Tennessee Self-concept Scale-2 (TSCS-2)	South Africa; Urban HIV clinics	Longitudinal study	Perinatally infected YLHIV, (N = 37)	Range: 9–14; Mean (SD): 11.6 (1.7)
Self-Concept	Tennessee Self-concept Scale-2 (TSCS-2)	South Africa; Urban Pediatric HIV clinics and public hospitals	RCT baseline data	PHIV, (N = 177)	Range: 9–14; Mean (SD): 11.68 (1.42)
Self-Efficacy	Self-Efficacy Questionnaire for Children	Tanzania; Urban weekly pediatric clinic	Pilot randomized waitlist-controlled trial	ALHIV, (N = 48)	Range: 14–18; Mean (SD): 15.7 (1.4)
Self-Efficacy	Self-Efficacy for Managing Chronic Disease 6-Item Scale (SE-6-Xhosa)	South Africa; Urban Community Health Centre	Cross-sectional	Xhosa women with HIV, (N = 229)	Range: 18–40; Mean (SD): 30.7 (4.8)
Self-Efficacy	HIV-Adherence self-efficacy assessment survey (HIV-ASES)	Kenya; Urba HIV care and treatment outpatient clinic	Cross-sectional	ALHIV, (N = 82)	Range: 16–19; Median (IQR): 17 (16–18)
Self-Efficacy	Ad hoc	South Africa: Urban primary schools	Mixed methods	Female adolescents, (N = 382)	Range: 11–16
Self-Efficacy	Ad hoc	South Africa: Urban primary care clinics	Pilot interventional study	Women living with HIV, (N = 120)	Range: 18–50; Mean (SD) Control: 28.4 (6.ca; Mean (SD) Intervention: 30.6 (5.8)
Self-Efficacy	Ad hoc	Eswatini: Rural and urban HIV care and treatment facilities	Cross-sectional	ALHIV, (N = 40)	Mean (SD): 15.5 (1.6)
Self-Efficacy	Ad hoc	Uganda; and Kenya; Facility-based	Cross-sectional	ALHIV, (N = 582)	Mean (SD): 14.6 (1.4)
Self-Esteem	Rosenberg Self-esteem Measure (RSEM-10)	Nigeria: Urban HIV support care center	Cross-sectional	HIV positive adults	Range: 18–62 Mean (SD): 30.9 (11.4)
Self-Esteem	Rosenberg Self-esteem Measure (RSEM-10)	Ghana; Urban Hospital-based clinic	Cross-sectional	PLHIV in Ghana & USA (N = 55 Ghana)	Range: 15–49
Self-Esteem	Rosenberg Self-esteem Measure (RSEM-10)	South Africa: Urban primary schools	Mixed methods	Female adolescents, (N = 382)	Range: 11–16
Self-Esteem	Rosenberg Self-esteem Measure (RSEM-10)	South Africa: Rural Community-based	Cross-sectional survey	YPLHIV, (N = 334)	Range: 12–24; Median (IQR): 21 (16–23)
Self-Esteem	Rosenberg Self-esteem Measure (RSEM-10)	Namibia; Rural health center	Exploratory design	PLHIV, (N = 124)	Range: 13–74; Mean (SD): 31.8 (10.9)
Self-Esteem	Rosenberg Self-esteem Measure (RSEM-10)	South Africa: Rural public healthcare facilities	Cross-sectional survey	YPLHIV, (N = 359)	Range: 12–24 Median (IQR): 21 (16–23)
Self-Esteem	Rosenberg Self-esteem Measure (RSEM-10)	South Africa: Urban	Cross-sectional	YPLHIV, (N = 76)	Range:15–24; Mean (SD): 19.4 (2.6)
Self-Esteem	Rosenberg Self-esteem Measure (RSEM-10)	Tanzania; Urban weekly pediatric clinic	Pilot randomized waitlist-controlled trial	ALHIV, (N = 48)	Range: 14–18 Mean (SD): 15.7 (1.4)
Self-Esteem	Rosenberg Self-esteem Measure (RSEM-10)	Ghana: Urban hospital	Cross-sectional study	ALHIV, (N = 139)	Range: 13–19; Mean (SD):16.6 (1.8)
Self-Esteem	Rosenberg Self-esteem Measure (RSEM-10)	Kenya; Urban- HIV care and treatment outpatient clinic	Cross-sectional	ALHIV, (N = 82)	Range 16–19; Median (IQR): 17 (16–18)
Self-Esteem	Rosenberg Self-esteem Measure (RSEM-10)	Uganda and Kenya: Facility-based	Cross-sectional	ALHIV, (N = 582)	Mean (SD): 14.6 (1.4)
Self-Esteem	Modified Rosenberg Self-esteem Measure (RSEM-8)	South Africa: Urban primary schools	Mixed methods	Female adolescents, (N = 382)	Range: 11–16
Self-Esteem	Tennessee Self-concept Scale-2 (TSCS-2)	Uganda; Rural public schools	Longitudinal study	AIDS orphaned adolescents, (N = 268)	Range: 11–17; Mean (SD): 13.7 (1.3)
Self-Esteem	Self-esteem-Hare Area-specific self-esteem scale	Uganda; Urban community clinic	Retrospective cohort study	Children with/ without perinatal HIV infection/ exposure, (N = 165)	Range: 6–18; Mean (SD): 10.8 (3.5)
Self-Esteem	Ad hoc	South Africa, Primary care clinics	RCT	Women living with HIV, (N = 143)	Range: 18–50
Self-Esteem	Ad hoc	Uganda and South Africa- Facility-based	Cross-sectional	Adults receiving palliative services, (N = 285)	Mean (SD): 40.1 (12.8)
Self-Worth	Ad hoc	Zimbabwe; Rural clinics	RCT	ALHIV, (N = 94)	Range: 10–15
Transcendence	Missoula Vitas Quality of Life Index (MVQOLI) Transcendent Subscale	Uganda and South Africa: Rural and Palliative Care Services	Cross-sectional	Adults receiving palliative services, (N = 285)	Mean (SD): 40.1 (12.8)

### Qualitative mapping of positive constructs

**Fig 2:**
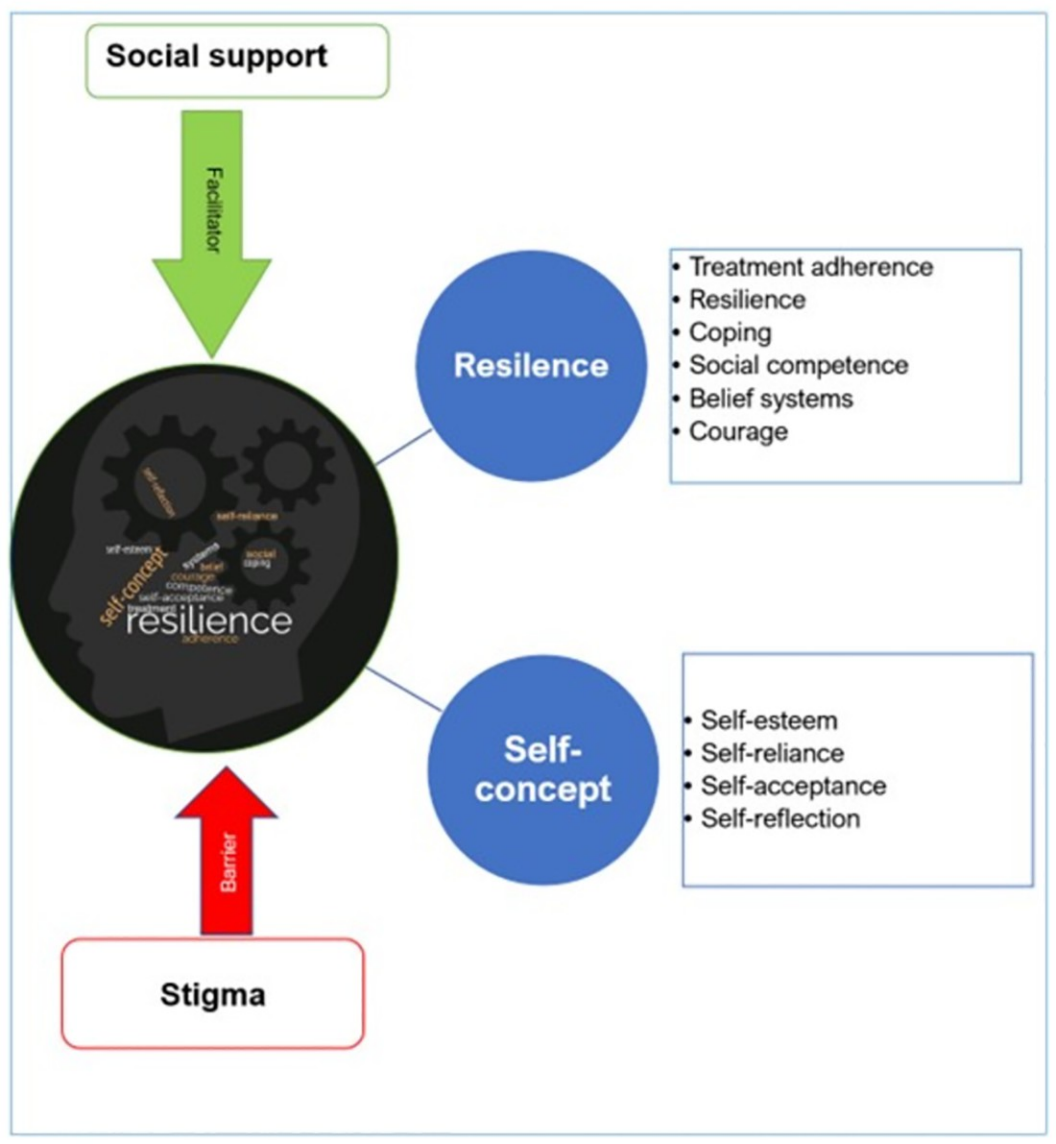
Qualitative mapping of positive psychological constructs.

From the qualitative studies, six positive psychological constructs emerged, i.e., courage, self-reliance, self-esteem, self-acceptance, resilience and coping (Fig 2). Self-concept was conceptualized as an interaction of self-esteem, self-reliance, self-acceptance and self-reflection. Self-concept is central to positive functioning; for example, high self-esteem is essential for living with HIV. The studies also suggested that resilience was crucial to coping with the demands of living with a chronic condition. Sociocultural belief systems shape resilience and are essential for treatment adherence. Social support optimized positive mental health function, with participants citing support from several sources (e.g., family and peers). Lastly, stigma and fear of disclosure were seen as the most significant barriers to positive psychological functioning—See S7 Table for further details.

### Quantitative mapping of positive constructs- Psychometric properties critical appraisal

#### Description of outcome measure characteristics

**Fig 3:**
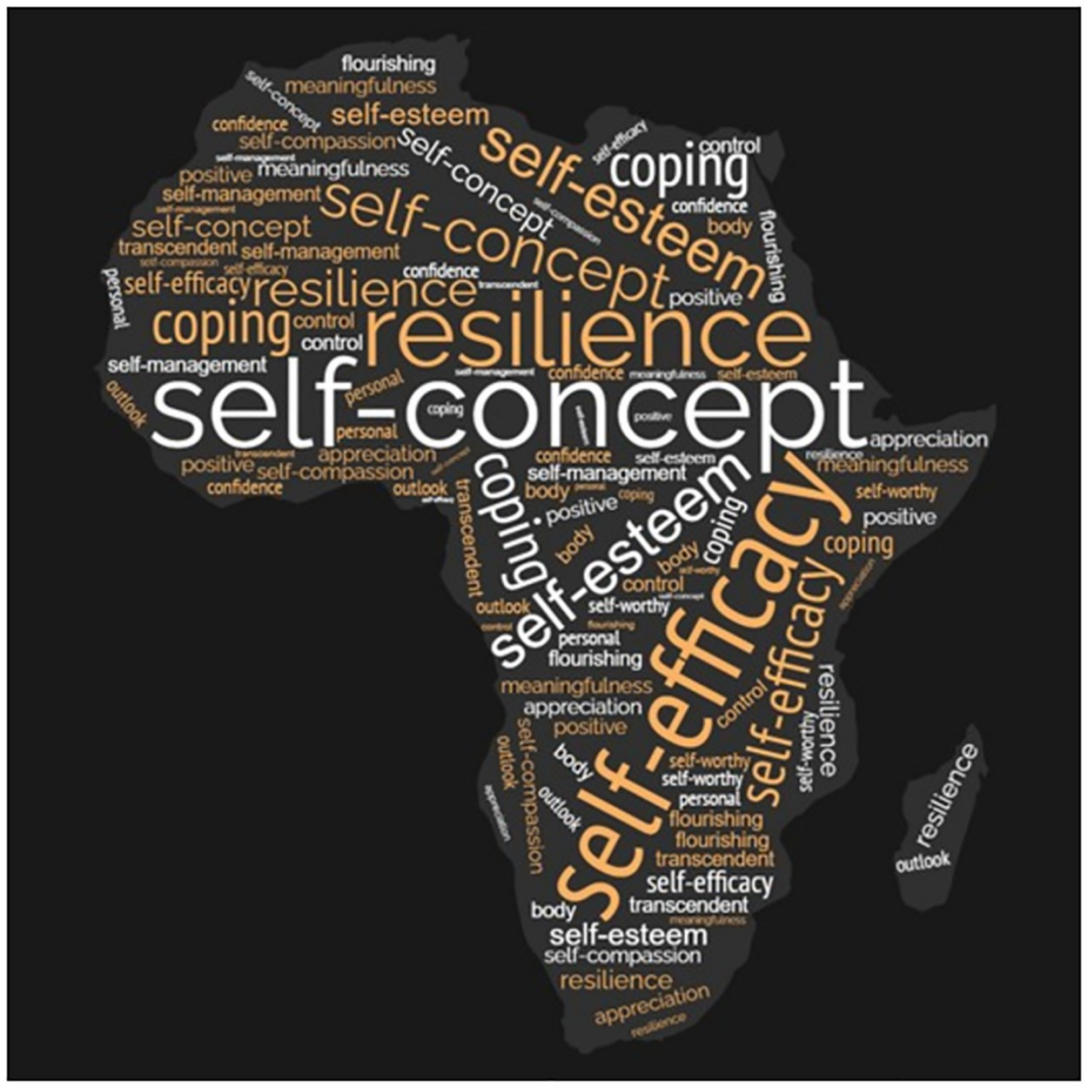
Constructs map.

We retrieved 36 outcome measures spanning 15 positive psychological constructs. Resilience, self-concept, self-esteem, coping and self-efficacy were the most reported constructs, as visually depicted in Fig 3. The item range for the outcomes was 5–45, with 19/36 (53%) scored on a 5-point Likert scale and most (29/36, 81%) available for free/without payment. However, only a few outcome measures (11/36, 31%) had scoring instructions—See S8 Table.

#### Results of individual outcomes sorted by construct

A description of the different outcome measures is presented subsequently; results are arranged alphabetically per construct. In Table 3, we present methodological quality/RoB assessment ratings, with Table 4 outlining the collation of quality of outcomes and best evidence synthesis.

**Table 3:** Methodological ratings.

Construct	Outcome Measure	Structural validity	Internal consistency	Convergent validity—comparison with other outcome measurement instruments	Discriminative or known-groups validity	Construct validity (hypotheses testing approach)—comparison with other outcome measurement instruments	Construct validity (hypotheses testing approach)—comparison between subgroups	Responsiveness- hypotheses testing before and after intervention
Body Appreciation	Body Appreciation Scale- 2 (BAS-2)			Adequate [32] ●only internal consistency was reported for the outcome measure				
Confidence	Ad hoc							Inadequate [33] ●No formal validation of outcomes despite pilot testing and adaptation
Coping	Psychological Adjustment to Illness Scale Self Report (PAIS-SR)			Inadequate [34] ●no translation and validation of outcomes for local use				Inadequate [34] ●sample too small for statistical tests
Coping	Acceptance of Illness Scale (AIS)			Doubtful [35] ●only the reliability coefficient reported				
Coping	Coping with HIV+ status						Inadequate [36] ●no psychometrics of outcome measures were provided	
Flourishing	Flourishing Well Being Scale (FBWS)			Doubtful [37] ●only the reliability coefficient reported				
Meaningfulness	HIV Meaningfulness Scale (HIVMS)			Doubtful [35] ●only the reliability coefficient reported				
Personal Control	Mastery Scale (MS)				Inadequate [38] ●sample size determined based on parameters from a Ghanaian study only ● Comparator outcomes not validated for local use			
Positive Outlook	Individual Protective Factors Index (IPFI)		Inadequate [39] ●Too small sample size for the pilot study (N = 15) to establish reliability indices ●No additional psychometrics were measured		Inadequate [39] ●Too small sample size for the pilot study (N = 15) to establish reliability indices ●No additional psychometrics were measured			
Resilience	Child Youth Resilience Measure-12			Doubtful [40] ● Cut-off points not stated ●Outcome measure not validated in study population not stated	Doubtful [40] ● Cut-off points not stated ●Outcome measure not validated in study population not stated			
Resilience	Connor-Davidson Resilience scale (CDRS-10)			Very Good l [41]				
Resilience	Connor-Davidson Resilience scale (CDRS-10)			Very Good [42]				
Resilience	Connor-Davidson Resilience scale (CDRS-10)			Very Good [37]				
Self-Management	Adolescent HIV Self-Management Scale (AdHIVSM)	Very good [43]		Doubtful [43] ● not all comparator instruments psychometrics are provided				
Self-Management	Adolescent HIV Self-Management Scale (AdHIVSM)		Very good [44]	Adequate [44] ● not all comparator instruments psychometrics are provided				
Self-Management	Adolescent HIV Self-Management Scale (AdHIVSM)	Very good [45]	Very good [45]	Adequate [45] ● not all comparator instruments psychometrics are provided				
Self-compassion	Self-compassion scale (SCS)			Doubtful [35] ●only the reliability coefficient reported				
Self-Concept	Beck Youth Self-Concept Scale (BYSCS)				Inadequate [46] ●no psychometrics for local adaptation	Inadequate [46] ●no psychometrics for local adaptation		
Self-Concept	Beck Youth Self-Concept Scale (BYSCS)				Inadequate [47] ●measurement properties not highlighted for all instruments. The cited papers do not have any psychometric data	Inadequate [47] ●measurement properties not highlighted for all instruments. The cited papers do not have any psychometric data		
Self-Concept	Beck Youth Self-Concept Scale (BYSCS)				Inadequate [48] ●measurement properties not highlighted for all instruments. The cited papers do not have any psychometric data	Inadequate [48] ●measurement properties not highlighted for all instruments. The cited papers do not have any psychometric data		
Self-Concept	Tennessee Self-concept (TSCS-2 20-item)			Doubtful [49] ●no psychometrics were provided, even in the referenced article			Doubtful [49] ●no psychometrics were provided, even in the referenced article	
Self-Concept	Tennessee Self-concept (TSCS-2 20-item)			Doubtful [50] ●Only Cronbach alpha is reported.				
Self-Concept	Tennessee Self-concept (TSCS-2 20-item)			Inadequate [51] ●Outcome not adapted and validated for local use. ●Only Cronbach alpha is reported				
Self-efficacy	Self-Efficacy Questionnaire for Children (SEQC)							Inadequate [52] ● tools were translated into the local language but were not validated.
Self-Efficacy	Self-Efficacy for Managing Chronic Disease 6-Item Scale (SE-6-Xhosa)			Inadequate [53] ●psychometrics not reported				
Self-Efficacy	Self-efficacy to protect oneself from unwanted sex							Inadequate [54] no details on validation of the adapted outcome measure.
Self-Efficacy	Self-efficacy against unwanted sex (SEPOUS)						Inadequate [36] ●no psychometrics of outcome measure	
Self-Efficacy	Self-efficacy for negotiating condom use (SENCU)				Inadequate [36] ●No psychometrics of outcome measure		Inadequate [36] ●no psychometrics of outcome measure	
	Self-efficacy to disclose HIV Questionnaire (SEDHQ)					Doubtful [55] ●no details of the scale development and validation process were provided. ●only Cronbach’s alpha indices were provided for comparator outcomes		
Self-esteem	Ad hoc						Inadequate [36] ●no psychometrics of outcomes	
Self-esteem	Ad hoc							Inadequate [33] ●No formal validation of outcomes despite pilot testing and adaptation
Self-esteem	Ad hoc		Inadequate [38] ●No psychometrics of outcomes					
Self-esteem	Self-esteem-Hare Area-specific self-esteem scale (HASSES)		Inadequate [39] ●Too small sample size for the pilot study (N = 15) to establish reliability indices ●No additional psychometrics were measured		Inadequate [39] ●Too small sample size for the pilot study (N = 15) to establish reliability indices ●No additional psychometrics were measured			
Self-esteem	Modified Rosenberg Self-esteem Measure (RSEM-8)							Inadequate [54] ●No details of the validation of the adapted Rosenberg- 8 scale
Self-esteem	Rosenberg Self-esteem Measure (RSEM-10)				Inadequate [38] ●outcome measure not validated in local setting (Ghana)			
Self-Esteem	Rosenberg Self-esteem Measure (RSEM-10)			Inadequate [56] ●outcome measure not validated in the local setting	Doubtful [56] ●Tools not validated			
Self-esteem	Rosenberg Self-esteem Measure (RSEM-10)					Doubtful [55] ●no psychometrics for local adaptation		
Self-esteem	Rosenberg Self-esteem Measure (RSEM-10)			Very good [42]				
Self-esteem	Rosenberg Self-esteem Measure (RSEM-10)							Inadequate [52] Tools were translated and not formally validated for use in the study population
Self-esteem	Rosenberg Self-esteem Measure (RSEM-10)			Inadequate [57] ●only Cronbach’s alpha scores presented for psychometrics & tools not adapted for local use				
Self-esteem	Rosenberg Self-esteem Measure (RSEM-10)			Inadequate [58] ● no information on cross-cultural adaptation & psychometrics	Inadequate [58] ● no information on cross-cultural adaptation & psychometrics			
Self-esteem	Rosenberg Self-esteem Measure (RSEM-10)			Inadequate [59] ● no information on cross-cultural adaptation & psychometrics				
Self-esteem	Rosenberg Self-esteem Measure (RSEM-10)			Very good [41]				
Self-Esteem	Rosenberg Self-esteem Measure (RSEM-10)			Adequate [32] ●Only construct validity and internal consistency were reported for some comparator outcome measures				
Self-worth	Ad hoc							Inadequate [33] ●No formal validation of outcomes despite pilot testing and adaptation
Transcendence	Missoula Vitas Quality of Life Index (MVQOLI) Transcendent Subscale				Moderate [60] ●No formal validation of translated outcome measures			

**Table 4:** Quality of psychometrics/evidence synthesis.

Construct	Outcome Measure	Structural validity	Internal consistency	Convergent validity—comparison with other outcome measurement instruments	Discriminative or known-groups validity	Construct validity (hypotheses testing approach)—comparison with other outcome measurement instruments	Construct validity (hypotheses testing approach)—comparison between subgroups	Responsiveness- hypotheses testing before and after intervention
Body Appreciation	Body Appreciation Scale 2 (BAS-2)			Methodology quality: Adequate Psychometric quality: + **Quality of evidence:** Moderate [32]				
Confidence	Ad hoc							Methodology quality: Inadequate Psychometric quality: + **Quality of evidence:** Very low [33]
Coping	Psychological Adjustment to Illness Scale Self Report (PAIS-SR)			Methodology quality: Inadequate Psychometric quality: + **Quality of evidence:** Very low [34]				Methodology quality: Inadequate Psychometric quality: + **Quality of evidence:** Very low [34]
	Acceptance of illness scale (AIS)			Methodology quality: Doubtful Psychometric quality: + **Quality of evidence:** Low [35]				
	Coping with HIV+ status						Methodology quality: Inadequate Psychometric quality: - **Quality of evidence:** Very low [36]	
Flourishing	Flourishing Well Being Scale (FBWS)			Methodology quality: Doubtful Psychometric quality: - **Quality of evidence:** Low [37]				
Meaningfulness	HIV Meaningfulness Scale (HIVMS)			Methodology quality: Doubtful Psychometric quality: + **Quality of evidence:** Low [35]				
Personal Control	Mastery Scale (MS)				Methodology quality: Inadequate Psychometric quality:? **Quality of evidence:** Very low [38]			
Positive Outlook	Individual Protective Factors Index (IPFI)		Methodology quality: Inadequate Psychometric quality: + **Quality of evidence**: Very low [39]		Methodology quality: Inadequate Psychometric quality: - **Quality of evidence:** Very low [39]			
Resilience	Child Youth Resilience Measure-12			Methodology quality: Doubtful Psychometric quality: + **Quality of evidence:** Low [40]	Methodology quality: Doubtful Psychometric quality: + **Quality of evidence**: Low [40]			
	CDRS-25			**Study 1** [41]**:** Methodology quality: doubtful Psychometric quality: + **Study 2** [42]**:** Methodology quality: very good Psychometric quality: - **Overall Quality of evidence:** Moderate				
	CDRS-10			Methodology quality: Doubtful Psychometric quality: - **Quality of evidence:** Low [37]				
Self-Management	Adolescent HIV Self-Management Scale (AdHIVSM)	**study 3:** [45] Methodology quality: Very good Psychometric quality**: +** **Study 1:**[43] Methodology quality: Inadequate Psychometric quality: - **Quality of evidence:** Very low	**Study 2** [44]**:** Methodology quality: Very good Psychometric quality: + **Study 3** [45]**:** Methodology quality: Very good psychometric quality: +	**Study 1:** [43] Methodology quality: Doubtful Psychometric quality: + **Study 2**: [44] Methodology quality: Adequate Psychometric quality:+ **Study 3** [45]Methodology quality: Adequate Psychometric quality**: +** **Quality of evidence:** Low				
Self-compassion	Self-compassion scale (SCS)			Methodology quality: Doubtful Psychometric quality: + **Quality of evidence:** Low [35]				
Self-Concept	Beck Youth Self-Concept Scale (BYSCS)			**Study 1** [46]**:** Methodology quality: Inadequate Psychometric quality: - **Study 2** [47]**:** Methodology quality: Inadequate Psychometric quality: - **Study 3** [48]**:** Methodology quality: Moderate Psychometric quality: + **Overall Quality of evidence:** Very low	**Study 1** [46]**:** Methodology quality: Inadequate Psychometric quality: + **Study 2** [47]**:** Methodology quality: Inadequate Psychometric quality:? **Study 3** [48]**:** Methodology quality: Inadequate Psychometric quality:+ **Overall Quality of Evidence:** Very low			
	Tennessee Self-concept Scale-2 (TSCS-2)			**Study 1** [49]**:** Methodology quality: Doubtful Psychometric quality: + **Study 2** [50]**:** Methodology quality: Doubtful Psychometric quality: + **study 3** [51] Methodology quality**:** Inadequate Psychometric quality:? **Quality of evidence:** Very low			Methodology quality: Doubtful Psychometric quality: - **Quality of evidence:** Very low [49]	
Self-efficacy	Self-Efficacy Questionnaire for Children (SEQC)							Methodology quality: Inadequate Psychometric quality: + **Quality of evidence:** Very low [52]
	Self-Efficacy for Managing Chronic Disease 6-Item Scale (SE-6-Xhosa)			Methodology quality: Inadequate Psychometric quality: + **Quality of evidence:** Very low [53]				
	Self-efficacy against unwanted sex (SEPOUS)							Methodology quality: Inadequate Psychometric quality: + **Quality of evidence:** Very low [54]
	Self-efficacy for correct condom use (SECCU)						Methodology quality: Inadequate Psychometric quality: - **Quality of evidence:** Very low [36]	
	Self-efficacy for negotiating condom use (SENCU)				Methodology quality: Inadequate Psychometric quality: - **Quality of evidence:** Very low [36]		Methodology quality: Inadequate Psychometric quality: - **Quality of evidence:** Very low [36]	
	Self-efficacy to disclose HIV Questionnaire (SEDHQ)					Methodology quality: Doubtful Psychometric quality: - **Quality of evidence: Very low** [55]		
Self-esteem	Rosenberg Self-esteem Measure (RSEM-10)			**Study 1** [38]**:** Methodology quality: Inadequate Psychometric quality: + **Study 2** [56]**:** Methodology quality: Inadequate Psychometric quality: + **Study 3** [57]**:** Methodology quality: Inadequate Psychometric quality: + **Study 4** [59]**:** Methodology quality: Inadequate Psychometric quality: + **Study 5**[42]**:** Methodology quality: Very good Psychometric quality: + **Study 6** [32]**:** Methodology quality: Adequate Psychometric quality: + **Study 7** [41]**:** Methodology quality: Very good Psychometric quality: + **Overall Quality of evidence:** Moderate	**Study 8** [58]**:** Methodology quality: Inadequate Psychometric quality:?	**Study 9** [55]**:** Methodology quality: Doubtful Psychometric quality: -	**Study 10** [52]**:** Methodology quality: Doubtful Psychometric quality: - **Quality of evidence:** Very low	
	Modified Rosenberg Self-esteem Measure (RSEM-8)							Methodology quality: Inadequate Psychometric quality: - **Quality of evidence:** Very low [54]
	Self-esteem-Hare Area-specific self-esteem scale (HASSES)		Methodology quality: Inadequate Psychometric quality: - **Quality of evidence:** Very low [39]		Methodology quality: Inadequate Psychometric quality: - **Quality of evidence:** Very low [39]			
	Ad hoc						Methodology quality: Inadequate Psychometric quality: + **Quality of evidence:** Very low [36]	
	Ad hoc		Methodology quality: Inadequate Psychometric quality: + **Quality of evidence:** Very low [38]					
	Ad hoc							Methodology quality: Inadequate Psychometric quality: + **Quality of evidence:** Very low [33]
Self-worth	Ad hoc							Methodology quality: Inadequate Psychometric quality: + **Quality of evidence**: Very low [33]
Transcendent	Missoula Vitas Quality of Life Index (MVQOLI) Transcendent Subscale				Methodology quality: Moderate Psychometric quality: + **Quality of evidence:** Moderate [61]			

#### Body appreciation

Body appreciation is defined as "accepting, holding favorable attitudes toward, and respecting the body, while also rejecting media-promoted appearance ideals as the only form of beauty" [62]. The Body Appreciation Scale-2 (BAS-2) was cited in one study [32]. There was moderate evidence of construct validity. The study was of moderate quality; only the internal consistency of the comparator outcomes was reported.

#### Confidence

Confidence can be defined as the belief in one’s capability to meet the demands of any task [62]. An ad-hoc confidence questionnaire measured confidence in one study [33]. There was very low evidence of construct validity; the study was of inadequate quality. Although the outcome was pilot-tested and adapted for local use with 10 participants, the outcome measure was not formally validated.

#### Coping

Coping is defined as "strategies, i.e., behaviors, skills or ways of regulating thoughts and emotions for dealing with stressors” [63]. Coping was reported in three studies; all used different outcome measures [34–36]. The Acceptance of Illness Scale (AIS) was cited in one study [35]. There was very low evidence of construct validity. The study was of inadequate quality; only the internal consistency of the comparator outcomes was reported, and the outcome measures were not translated and validated for local use.

An ad hoc questionnaire was used to measure coping with HIV in one study [36]. There was very low evidence of construct validity. The study was of inadequate quality; no psychometrics of the comparator outcomes were reported, and the outcome measures were not translated and validated for local use. The Psychological Adjustment to Illness Scale Self Report (PAIS-SR) was cited in one study [34]. There was very low evidence of construct validity and responsiveness. The study was of inadequate quality; only the internal consistency of the comparator outcomes was reported. Also, the outcome measures were not translated and validated for local use. Lastly, inappropriate tests were used for analysis to measure responsiveness; t-tests were used for a very small sample (N = 19).

#### Flourishing

Flourishing can be defined as "a combination of feeling good and functioning effectively and is synonymous with a high level of mental well-being" [64]. The Flourishing Well Being Scale (FWBS) was cited in one study [37]. There was moderate evidence of construct validity. The study was of very good quality, and comparator outcome measures were translated and validated in the research setting. However, the study produced null findings; flourishing was equitable for those on Antiretroviral therapy (ART) and those not on ART.

#### Meaningfulness

Meaningfulness is "the cognizance of order, coherence and purpose in one’s existence, the pursuit and attainment of worthwhile goals and an accompanying sense of fulfillment" [65]. The HIV Meaningfulness Scale (HIVMS) was cited in one study [35]. There was very low evidence of construct validity. The study was of inadequate quality; only the internal consistency of the comparator outcomes was reported. Further, the outcome measures were not translated and validated for local use.

#### Personal control

Personal control can be defined as "…a learned repertoire of goal-directed skills that enable humans to act upon their aims, postpone gratification and overcome difficulties relating to thoughts, emotions and behaviors" [66]. The Mastery Scale (MS) was cited in one study to measure personal control [38]. There was very low evidence of construct validity. The study was of inadequate quality; the outcome measures were not translated and validated for local use. Also, the study sample size was determined based on parameters from a Ghanaian target sample, yet the study compared outcomes across Ghanaian and US participants.

#### Positive outlook

A positive outlook can be defined as optimism about a great future with or without experiencing adverse events [66]. The Individual Protective Factors Index (IPFI) was cited in one study [39]. There was very low evidence of internal consistency and construct validity. The study was of inadequate quality. Although the outcomes were translated and adapted for use in Uganda, the investigators used too small a sample for the pilot study (N = 15) to establish reliability indices. Further, no additional psychometrics were measured for the adapted outcome measures.

#### Resilience

Resilience is the ability to bounce back from adverse circumstances and maintain optimal mental health functioning [67]. Resilience was evaluated in four studies. One study used the Child Youth Resilience Measure-12 (CYRM-12) [40], with three studies using the Connor-Davidson Resilience scale (CDRS-10) [37, 41, 42]. The CYRM-12 was cited in one study [40]. There was very low evidence of construct validity. The study was of doubtful quality; the outcome measure was not translated and validated for local use. There was high evidence of construct validity of the CDRS-10. All three studies were of very good quality [37, 41, 42], with the CDRS-10 previously translated and validated in South African adolescents [68].

#### Self-management

Self-management can be defined as the ability to take necessary steps, including adhering to treatment regimens in managing a condition [43, 44]. The Adolescent HIV Self-Management Scale (AdHIVSM) was cited in three studies [43–45]. There was high evidence of content validity [45], structural validity [43, 45] and internal consistency [44, 45]; the studies were of very good quality. There was moderate evidence of construct validity: not all the psychometrics of the comparator outcomes were reported [43, 44].

#### Self-compassion

Self-compassion can be defined as "*being open to and moved by one’s own suffering*, *experiencing feelings of caring and kindness toward oneself*, *taking an understanding*, *non-judgmental attitude toward one’s inadequacies and failures*, *and recognizing that one’s experience is part of the common human experience"* [69]. The Self-Compassion Scale (SCS) was cited in one study [35]. There was very low evidence of construct validity. The study was of inadequate quality; only the internal consistency of the comparator outcomes was reported, and the outcome measures were not translated and validated for local use.

#### Self-concept

Self-concept can be defined as how someone perceives and evaluates themselves relative to peers [70]. Six studies evaluated self-concept using the Beck Youth Self-Concept Scale (BYSCS) [46–48] and the Tennessee Self-Concept Scale [49–51]. The BYSCS was cited in three studies [46–48]. There was very low evidence of construct validity. The three studies were of inadequate quality. Although the secondary outcome measures were translated into the local languages, they were not fully validated. Also, the cross-referenced articles did not contain the validation data cited by the authors but rather, generic statements on translating the outcomes [48, 71]. The Tennessee Self-concept Scale-2 (TSCS-2) was cited in three studies [49–51]. There was very low evidence of construct validity. The three studies were of inadequate quality. Although the secondary outcome measures were translated into the local language, they were not validated. Also, the cross-referenced articles did not contain the validation data as cited by the authors [49, 51].

#### Self-efficacy

Self-efficacy is defined as self-belief in the capability to execute a specific task regardless of the magnitude of potential obstacles [70]. We analyzed six variants of self-efficacy outcome measures reported across five studies [36, 52–55]. An ad hoc self-efficacy for correct condom use (SECCU) questionnaire was used to measure self-efficacy for correct condom use in a single study [36]. There was very low evidence of construct validity. The study was of inadequate quality; no psychometrics of the comparator outcomes were reported, and the outcome measures were not translated and validated for local use. An ad hoc self-efficacy for negotiating condom use (SENCU) questionnaire was used to measure self-efficacy for negotiating condom use in a study [36]. There was very low evidence of construct validity. The study was of inadequate quality. No psychometrics of the comparator outcome measures were reported. Also, the outcome measures were not translated and validated for local use. The Self-Efficacy Questionnaire for Children (SEQC) was cited in one study [52]. There was very low evidence of construct validity. The study was of inadequate quality. Although the outcomes were translated into the local language, they were not formally validated.

The Self-Efficacy for Managing Chronic Disease 6-Item Scale (SE-6-Xhosa) was cited in one study [53]. There was very low evidence of construct validity. The study was of inadequate quality. There was no transcultural adaptation, and no psychometrics were reported. The Self-efficacy to protect oneself from unwanted sex (SEPOUS) was cited in one study [54]. There was very low evidence of responsiveness. The study was of inadequate quality; no psychometrics were reported, including the transcultural adaptation of comparator outcome measures. The study purpose-built SEDHQ Self-efficacy to disclose HIV Questionnaire (SEDHQ) was used in one study [55]. There was very low evidence of responsiveness. The study was of inadequate quality; no scale development and validation process details were provided. Also, only Cronbach’s alpha indices were provided for comparator outcomes.

#### Self-esteem

Self-esteem can be defined as "… as an attitude toward one’s self—based on one’s feelings of worth as a person" [72]. Self-esteem was measured using six different outcome measures, i.e., three ad hoc questionnaires [33, 36, 38], Hare Area-specific self-esteem scale (HASSES), Rosenburg Self-esteem Measure (RSEM-10) [32, 38, 41, 42, 52, 55–59] and the Modified Rosenburg Self-esteem Measure (RSEM-8) [39]. Study purpose-built (ad-hoc) self-esteem questionnaires were used in three studies [33, 36, 38]. There was very low evidence of construct validity. The studies were of inadequate quality; no details were provided for developing and validating the ad hoc measures.

#### Hare Area-specific Self-esteem Scale (HASSES)

The HASSES was cited in one study [39]. There was very low evidence of internal consistency and construct validity. The study was of inadequate quality. Although the outcomes were translated and adapted for use in Uganda, the investigators used too small a sample for the pilot study (N = 15) to be able to establish reliability indices. Further, no additional psychometrics were measured for the adapted outcome measures.

The Rosenberg Self-esteem Measure (RSEM-10) was used in 10 studies of varying methodological quality. There was moderate evidence for construct validity. The methodological ratings were: very good [41, 42], adequate [32] doubtful [55, 59] and inadequate [38, 52, 56–58]. The methodological down gradings for the construct validity evaluation studies were mainly due to the lack of reporting of psychometrics; cross-cultural adaptation was not performed in most of the studies [38, 52, 56, 57]. There was high evidence of known-group validity from a single study of very good methodological quality [42]. The Modified Rosenberg Self-esteem Measure (RSEM-8) was cited in one study [54]. There was very low evidence of responsiveness. The study was of inadequate quality. Two items were omitted from the original RSEM-10, but the transcultural adaptation process details were not provided. Also, no psychometrics were reported, including those of comparator outcome measures.

#### Self-worth

Self-worth can be defined as "…an individual’s evaluation of himself or herself as a valuable, capable human being deserving of respect and consideration" [72]. A study used an ad hoc questionnaire to measure self-worth [33]. There was very low evidence of construct validity. The study was of inadequate quality. Although the outcome was pilot-tested and adapted for local use on 10 participants, the outcome measure was not formally validated.

#### Transcendence

Transcendence can be defined as "…a state of existence or perception that is not definable in terms of normal understanding or experience" [61]. The Missoula Vitas Quality of Life Index (MVQOLI) Transcendence Subscale was cited in one study [60]. There was moderate evidence of construct validity. The solitary study was of moderate quality; only the internal consistency of the comparator outcomes was reported.

## Discussion

This review sought to identify positive psychological outcomes used in AYALHIV in SSA, map the constructs onto corresponding measures, and critically appraise the identified outcomes’ psychometrics. We gleaned 15 positive psychological constructs, namely body appreciation, confidence, coping, flourishing, meaningfulness, personal control, positive outlook, resilience, self-management, self-compassion, self-concept, self-efficacy, self-esteem, self-worth and transcendence. Resilience, self-efficacy and self-esteem were the most measured constructs. Construct validity and internal consistency were the most measured properties, with content and structural validity being the least measured psychometrics. The implications of the individual measurement properties are discussed subsequently.

### Qualitative mapping of positive psychological constructs

In our study, social support was used as an umbrella term to encompass the types of support that occurred at family, peer and community levels. At the interpersonal level, family and peer support assisted adolescents in coping with negative feelings and facilitating belongingness [61, 73]. Peer social support helped ALHIV achieve the goals of giving and receiving social support, gaining health and relationship advice, adhering to healthcare regimens, learning practical skills and enjoying recreational activities as a group [74]. Social support has also precipitated a positive outlook of life among ALHIV, with adolescents believing that people living with HIV should be allowed to marry and have children if they so desire. Correspondingly, previous studies have shown that adolescents reporting a lack of social support and strained social and interpersonal relations also reported neglect, differential treatment, mistreatment [75] and a decreased sense of belonging [22]. The intersection between social support and stigma becomes more evident at the community level. Adolescents fear disclosing their status because they fear stigmatization, ridicule, gossip and insults within the school and community [75, 76].

Robust self-esteem among adolescents living with HIV enabled them to overcome stigma, increase their self-reliance and accept their HIV status [77]. Resilience is shaped by cultural and religious beliefs and the capacity to self-reflect and face adverse conditions [77, 78]. Resilient adolescents had greater life satisfaction [78] and were accepting of their circumstances. Resilience was vital for young persons to cope with their realities [77] and muster the courage to face possible stigma [75]. Among adolescents, disclosure to others was not always based on choice; the process is emotional and complex, with uncertain outcomes [79]. However, disclosure positively influenced adherence and retention to care, health improvement, and enabled social participation [80].

### Structural validity

Despite the wide use of positive psychological measures, the evidence for structural validity was limited. Except for the Adolescent HIV Self-Management Scale [35], all outcome measures analyzed were developed in high-income settings but were not properly translated and validated before use in the sub-Saharan region. Robust transcultural adaptations are essential for preserving structural validity, a fundamental psychometric property [81–83]. Structural/factorial validity measures the extent to which items measure the latent constructs purportedly measured by a specific outcome measure [81]. For example, the Connor-Davidson Resilience scale was the most commonly used resilience outcome measure, applied in three of the five resilience studies [37, 41, 42]. Yet none of these studies evaluated structural validity. Also, the Rosenburg Self-esteem Scale was used in ten studies in which we analyzed and assessed self-esteem [38, 41, 42, 52, 55–57, 59] but no study evaluated its structural validity.

Further, an outcome measure may perform differently when applied to two different geographical regions in the same country owing to sociocultural and linguistic differences. For instance, the Flourishing Scale exhibited measurement invariance/differential item functioning in South African university students [84]. Three of the eight items performed differently across the study’s four languages, i.e., English, Afrikaans, Sesotho and Setswana [84]. Robust transcultural translations and adaptations, including structural validity assessment, are essential before using an outcome measure with seemingly high psychometric robustness in another country. Psychometric performance in another country can never be assumed.

During the analysis, we observed a trend of snowball citations, i.e., the tendency to cite previously published studies to justify the validity of applied outcome measures [85]. Snowball referencing is problematic as the actual measurement properties of most positive psychological outcomes remain elusive. For example, a Kenyan study explored the construct validity of the Rosenberg Self-esteem Scale by investigating the correlates of self-esteem to self-efficacy in HIV treatment adherence as measured by the ART Adherence Self-efficacy (HIV-ASES) [56]. The study by Gitahi-Kamau et al. (2022) cites a previous validation study performed in the US as evidence of the psychometric robustness of the HIV-ASES [86]. However, the HIV-ASES did not undergo transcultural adaptation and validation before use in Kenya; this may lead to measurement bias.

### Construct validity

Construct validity is the extent to which scores on two outcomes correlate [81]. Sufficient evidence of structural validity is a prerequisite for construct validity [81]. In this review, construct validity was the most measured psychometric, with most tools showing evidence of moderate to high collective robustness. The high construct validity evidence across outcome measures may indeed imply that the outcomes were measuring what they were intended to measure. However, the lack of structural validity may "invalidate" evidence of construct validity robustness [81]. This contradiction (lack of structural validity) poses a measurement error dilemma, as most outcomes are still performed satisfactorily. Most of the outcomes had positive ratings regarding the quality of construct validity. The Pearson correlation coefficient was the most applied bivariate correlation index. The robustness of the Pearson correlation is a function of normality and sample size. None of the studies reported normality indices. However, most studies recruited samples ≥100; with a larger sample size, weak correlations are likely to show statistical significance. That said, the correlations were in the moderate ranges, with only two studies yielding robust correlations, i.e., R≥0.8; this may downgrade the overall evidence of construct validity. Nevertheless, building on the strong construct validity evidence across outcomes, there is a need for solid efforts for proper validation, i.e., measuring both structural and construct validity to ensure measurement equivalency to facilitate cross-cultural comparisons [81–83].

### Responsiveness

Responsiveness is the ability of an outcome measure to detect change over time [81]. There was insufficient evidence of responsiveness across the outcomes. Despite pilot testing and adaptation, none of the studies evaluating responsiveness formally validated outcomes before use [33, 52, 54]. In some instances, no psychometrics were provided [54]. For example, a randomized controlled trial evaluated the effectiveness of a peer-led intervention in improving linkage to and retention in care, adherence to ART, and psychosocial well-being among adolescents living with HIV in rural Zimbabwe [33]. The study applied an ad-hoc questionnaire measuring confidence, self-esteem and self-worth as secondary outcomes. Although the positive psychological outcome measures were translated into the native language and pilot-tested, the tools were not formally validated. The study demonstrated intervention effectiveness on the primary outcome (viral load suppression) and positive psychological constructs. It is reasonable to scale up the intervention at the clinical level. However, this may be problematic when inferring the intervention effects on the measured positive psychological outcomes. Using unvalidated outcomes may distort intervention effect sizes, which may lead to incorrect conclusions. More efforts are required to ensure that positive outcomes are adequately validated, with norms or cut-off scores identified before the measures are used for intervention(s) evaluation.

### Reliability

Reliability measures the extent of stability and reproducibility of outcomes, assuming constancy in extraneous variables [81, 87]. The Cronbach alpha was the most cited reliability index, with overall evidence of reliability in the moderate to high range. As observed in previous reviews, the Cronbach Alpha was inappropriately used in most studies to indicate psychometric robustness [85, 88, 89]. There were instances where outcome measures were adapted and translated, with the Cronbach alpha cited as evidence of reliability and validity. The Cronbach alpha measures the degree of items’ connectedness; it is neither a true indicator of internal consistency, a form of reliability, nor of validity [81, 87, 90]. Evaluation of Cronbach alpha is not a substitute for full validation. Compared to other forms of reliability, such as test-retest reliability and split-half reliability indexes, Cronbach alpha is the "least desired/robust" reliability indicator [87, 90]. Although most outcomes yielded high Cronbach alphas, there is a need for properly designed and fully powered psychometric evaluation studies. For instance, structural validity must be established before evaluating internal consistency and construct validity [81].

### Clinical and research utility

All but three outcome measures were available free of charge; this increases the utility of the identified positive psychological outcome measures. Most outcomes were rated on 4- or 5-point Likert scales, with some using 7-point Likert scales. Consideration must be made during transcultural adaptations to ensure age- and developmental-appropriate adaptations. For example, it is essential to decrease the number of response options to increase the feasibility of use in AYALHIV. For instance, HIV-related neurological impairment can reduce AYALHIV’s cognitive capabilities. In Africa, more common lower levels of education and HIV itself can impede school attendance and learning in this context. A previous validation study in Uganda had to collapse seven response options to five in addition to using visual cues as participants had difficulties understanding the original scoring instructions [91]. Cultural and linguistic differences must be accounted for to ensure equivalence between the original and target languages [82]. Robust transcultural translations and adaptations are critical, given that most of the outcomes gleaned were adopted from high-income countries. Most of the outcome measures were brief; this decreases respondent burden and increases the feasibility of research and routine use for evidence-based care. Also, most of the outcomes were generic; this allows comparisons across conditions and settings, and could be applicable for use in other chronic diseases. However, very few tools had established cut-off points; this makes comparisons across studies and contexts difficult. Overall, most of the tools had a high utility for routine use, given that most were generic brief, had fewer response options, and were available at low or no cost [30].

### Limitations

A significant limitation of the current review is that most studies analyzed were not primarily psychometric evaluation studies. As such, the odds of high risk of bias (RoB) ratings were great, given that the COSMIN checklist, which we utilized to evaluate methodological quality, was primarily designed to appraise psychometrics evaluation studies. For example, there was poor evidence of responsiveness across the outcome measures analyzed. None of the analyzed studies were primarily designed to evaluate responsiveness. Instead, we analyzed results from interventional studies to assess responsiveness. The COSMIN checklist is considered a "gold standard" for RoB evaluations but has limitations; it overtly gravitates to the stringent spectrum of psychometric RoB checklists [92]. However, we utilized multiple methods to ensure fair judgments per study. For instance, we contacted authors to get information essential for RoB ratings, which may not have been published to avoid reporting bias. Also, we had consensus meetings to synthesize all findings, as the first round of RoB ratings had yielded poor ratings for most outcomes. Due to resource limitations, the systematic review only included peer-reviewed articles published in English; this may have introduced language bias. Additionally, this may have limited our reach. Nevertheless, applying the PRISMA guidelines throughout increases the robustness of the review findings despite the inevitable methodological pitfalls.

## Conclusion

We identified 15 positive psychological constructs applied in AYALHIV in SSA: body appreciation, confidence, coping, flourishing, meaningfulness, personal control, positive outlook, resilience, self-management, self-compassion, self-concept, self-efficacy, self-esteem, self-worth and transcendence. Of the identified outcome measures, the Rosenberg Self-esteem Measure (RSEM-10), Missoula Vitas Quality of Life Index Transcendent Subscale, the Adolescent HIV Self-Management Scale, Connor-Davidson Resilience scale, Flourishing Well-Being Scale, and the Body Appreciation Scale-2 had moderate to high evidence of psychometric robustness. We recommend these outcome measures for routine research and clinical use. Also, further psychometric evaluation is warranted, and efforts should be made to produce shorter versions of some of the measures (e.g., Missoula Vitas Quality of Life Index Transcendent Subscale, the Adolescent HIV Self-Management Scale), for routine clinal use. Few studies performed complete validations; thus, evidence for psychometric robustness was fragmented. However, this review demonstrates the initial evidence of the feasibility of positive psychological outcomes for use in AYALHIV in low-resource settings. Instead of creating new outcomes, authors are advised to leverage the existing outcomes, adapt them for use and, if appropriate, strive to maintain the factorial structure to facilitate comparisons. Lastly, validating composite positive psychological outcomes should be considered for transcultural adaptations, given the variable psychometric performance across constructs and measurement properties.

## Supporting information

S1 Table Preferred Reporting Items for Systematic reviews and Meta-Analyses extension for Scoping Reviews (PRISMA-ScR) checklist. (DOCX)

S2 Table PRISMA checklist. (DOCX)

S3 Table CINAL search strategy. (DOCX)

S4 Table Operational definitions of psychometric properties. (DOCX)

S5 Table Updated criteria for good measurement properties. (DOCX)

S6 Table GRADE checklist- best evidence synthesis. (DOCX)

S7 Table Qualitative mapping of psychological constructs. (DOCX)

S8 Table Outcomes utility. (DOCX)
