# Supplementary material for: A conceptualization and psychometric evaluation of positive psychological outcome measures used in adolescents and young adults living with HIV: A mixed scoping and systematic review
Source: PLOS Glob Public Health. 2024 Aug 12;4(8):e0002255. doi: 10.1371/journal.pgph.0002255 (PMC11318935; doi:10.1371/journal.pgph.0002255)
Supplement: S3 Table — (DOCX) [file pgph.0002255.s003.docx]

## S3 Table: CINAL search strategy

| **Search #** | **Query** |
| --- | --- |
| **1** | **adolescen* OR juvenile* OR teen* OR youth* OR “young person*” OR “young people” OR Adolescence OR Young Adult** |
| 2 | Hiv OR hiv-1* OR hiv-2*OR hiv1 OR hiv2 OR “HIV infect*” OR “human immunodeficiency virus” OR “human immunodeficiency virus” OR “human immuno-deficiency virus” OR “human immune-deficiency virus” OR “acquired immunodeficiency syndrome” OR “acquired immunodeficiency syndrome” OR “acquired immuno-deficiency syndrome” OR “acquired immune-deficiency syndrome” OR HIV Infections OR Human Immunodeficiency Virus |
| 3 | “human immun*” AND “deficiency virus” |
| 4 | hiv or aids or acquired human immunodeficiency syndrome or human immunodeficiency virus OR HIV/AIDS |
| 5 | “acquired immun*” AND “deficiency syndrome” |
| **6** | **2 OR 3 OR 4 0R 5** |
| 7 | hope* OR optimis* OR resilien* OR cope OR coping OR gratitude OR grateful OR happiness OR joy OR gladness OR satisf* OR “self efficacy” OR self-efficacy OR content*OR wellbeing OR well-being OR “self acceptance” OR self-acceptance OR “self esteem” OR self-esteem OR “self concept” OR self-concept OR “self confidence” OR self-confidence OR “self perception” OR self-perception OR “self worth” OR self-worth OR “personal growth” OR tranquil* OR perseverance OR vitality OR meaning OR “social* inclus*” OR “social participation” OR “social engagement” OR “social support” OR “self care” OR self-care OR “positive attitude” OR “positive thinking” OR “positive mindset” OR mindfulness OR empower*OR love OR spiritual* OR “community integration” OR “community participation” OR humour OR dignity OR pleasure OR creativ* OR transcend* OR goal* OR Psychological Well-Being OR Hope OR Optimism OR Coping OR Human Dignity OR Hardiness OR Adaptation, Psychological OR Happiness OR Personal Satisfaction OR Self Efficacy OR Self Concept OR Social Inclusion OR Social Participation OR Self Care OR Mindfulness OR Empowerment OR Love OR Spirituality OR Social Networks OR Pleasure OR Creativeness OR Self Transcendence OR (Goals and Objectives) |
| 8 | hope* OR optimis* OR optimism OR resilience OR resilien* OR resilient OR cope OR coping OR gratitude OR grateful OR happiness OR joy OR gladness OR (life satisfaction) OR satisfaction OR satisf* OR “self efficacy” OR self-efficacy OR content OR contentment OR content*OR wellbeing OR well-being OR “self acceptance” OR self-acceptance OR “self esteem” OR self-esteem OR “self concept” OR self-concept OR “self confidence” OR self-confidence OR “self perception” OR self-perception OR “self worth” OR self-worth OR “personal growth” OR tranquillity OR tranquil* OR perseverance OR vitality OR meaning OR “social* inclus*” OR “social participation” OR “social engagement” OR “social support” OR “self care” OR self-care OR “positive attitude” OR “positive thinking” OR “positive mindset” OR mindfulness OR empowerment OR empower*OR love OR spirituality OR spiritual* OR “community integration” OR “community participation” OR humour OR dignity OR pleasure OR creativ* OR transcend* OR goal* OR Psychological Well-Being OR Hope OR Optimism OR Coping OR Human Dignity OR Hardiness OR Adaptation, Psychological OR Happiness OR Personal Satisfaction OR Self Efficacy OR Self Concept OR Social Inclusion OR Social Participation OR Self Care OR Mindfulness OR Empowerment OR Love OR Spirituality OR Social Networks OR Pleasure OR Creativeness OR Self Transcendence OR (Goals and Objectives) OR growth mindset OR positive mindset OR (*mindset) OR flourishing OR flourish* OR thriving OR thriv* |
| **9** | **7 OR 8** |
| 10 | Angola or Benin or Botswana or Burkina Faso or Burundi or Cameroon or Cape Verde or Central African Republic or CHAD or Comoros or Congo or Congo Democratic Republic or Djibouti or Equatorial Guinea or Eritrea or Ethiopia or Gabon or Gambia or Ghana or Guinea or Guinea-Bissau or Cote d'Ivoire or Ivory Coast or Kenya or Lesotho or Liberia or Madagascar or Malawi or Mali or Mozambique or Namibia or Niger or Nigeria or Sao tome and Principe or Rwanda or Senegal or Seychelles or Sierra Leone or Somalia or South Africa or South Sudan or Sudan or Swaziland or Tanzania or Togo or Uganda or Zambia or Zimbabwe |
| 11 | ‘Africa, South of the Sahara’ OR “sub-Saharan Africa” |
| **12** | **10 OR 11** |

**Adapted from* [18]
