# Supplementary material for: A conceptualization and psychometric evaluation of positive psychological outcome measures used in adolescents and young adults living with HIV: A mixed scoping and systematic review
Source: PLOS Glob Public Health. 2024 Aug 12;4(8):e0002255. doi: 10.1371/journal.pgph.0002255 (PMC11318935; doi:10.1371/journal.pgph.0002255)
Supplement: S4 Table — (DOCX) [file pgph.0002255.s004.docx]

## S4 Table: Operational definitions of psychometric properties [22,23]

| **Term** | | | **Definition** |
| --- | --- | --- | --- |
| **Domain** | **Measurement property** | **Aspect of a measurement**  **property** |  |
| **Reliability** |  |  | The degree to which the measurement is free from measurement error |
| **Reliability (extended definition)** |  |  | The extent to which scores for patients who have not changed are the same for repeated measurement under several conditions: e.g., using different sets of items from the same PROM (internal consistency); over time (test‐retest); by different persons on the same occasion (inter‐ rater); or by the same persons (i.e., raters or responders) on different occasions (intra‐rater) |
|  | **Internal consistency** |  | The degree of the interrelatedness among the items |
|  | **Reliability** |  | The proportion of the total variance in the measurements which is due to 'true’† differences  between patients |
|  | **Measurement error** |  | The systematic and random error of a patient's score that is not attributed to true changes in the  construct to be measured |
| **Validity** |  |  | The degree to which a PROM measures the construct(s) it purports to measure |
|  | **Content validity** |  | The degree to which the content of a PROM is an adequate reflection of the construct to be measured |
|  |  | **Face validity** | The degree to which (the items of) a PROM indeed looks as though they are an adequate reflection of the construct to be measured |
|  | **Construct validity** |  | The degree to which the scores of a PROM are consistent with hypotheses *(for instance with regard to internal relationships, relationships to scores of other instruments, or differences between relevant groups)* based on the assumption that the PROM validly measures the construct to be measured |
|  |  | **Structural validity** | The degree to which the scores of a PROM are an adequate reflection of the dimensionality of the construct to be measured |
|  |  | **Hypotheses testing** | Item construct validity |
|  |  | **Cross-cultural validity** | The degree to which the performance of the items on a translated or culturally adapted PROM are an adequate reflection of the performance of the items of the original version of the PROM |
|  | **Criterion validity** |  | The degree to which the scores of a PROM are an adequate reflection of a 'gold standard' |
| **Responsiveness** |  |  | The ability of a PROM to detect change over time in the construct to be measured |
|  | **Responsiveness** |  | Item responsiveness |
| **Interpretability*** |  |  | Interpretability is the degree to which one can assign qualitative meaning ‐ that is, clinical or commonly understood connotations – to a PROM's quantitative scores or change in scores. |

*† The word 'true' must be seen in the context of the CTT, which states that any observation is composed of two components – a true score and error associated with the observation. 'True' is the average score that would be obtained if the scale were given an infinite number of times. It refers only to the consistency of the score, and not to its accuracy.*

** Interpretability is not considered a measurement property, but an important characteristic of a measurement instrument*
