# Supplementary material for: A conceptualization and psychometric evaluation of positive psychological outcome measures used in adolescents and young adults living with HIV: A mixed scoping and systematic review
Source: PLOS Glob Public Health. 2024 Aug 12;4(8):e0002255. doi: 10.1371/journal.pgph.0002255 (PMC11318935; doi:10.1371/journal.pgph.0002255)
Supplement: S5 Table — (DOCX) [file pgph.0002255.s005.docx]

## S5 Table: Updated criteria for good measurement properties [19,20]

| **Measurement property** | **Rating1** | **Criteria** |
| --- | --- | --- |
| Structural validity | **+** | **CTT:**  CFA: CFI or TLI or comparable measure >0.95 OR RMSEA  <0.06 OR SRMR <0.082  **IRT/Rasch**:  No violation of unidimensionality3: CFI or TLI or comparable measure >0.95 OR RMSEA <0.06 OR SRMR <0.08  *AND*  no violation of local independence: residual correlations among the items after controlling for the dominant factor < 0.20 OR Q3's < 0.37  *AND*  no violation of monotonicity: adequate looking graphs OR item scalability >0.30  *AND*  adequate model fit:  IRT: χ2 >0.01  Rasch: infit and outfit mean squares ≥ 0.5 and ≤ 1.5 OR Z‐ standardised values > ‐2 and <2 |
|  | **?** | CTT: Not all information for '+' reported IRT/Rasch: Model fit not reported |
|  | **–** | Criteria for '+' not met |
| Internal consistency | **+** | At least low evidence4 for sufficient structural validity5 AND Cronbach's alpha(s) ≥ 0.70 for each unidimensional scale or subscale6 |
|  | **?** | Criteria for "At least low evidence4 for sufficient structural validity5" not met |
|  | **–** | At least low evidence4 for sufficient structural validity5 AND Cronbach’s alpha(s) < 0.70 for each unidimensional scale or subscale6 |
| Reliability | **+** | ICC or weighted Kappa ≥ 0.70 |
|  | **?**  **–** | ICC or weighted Kappa not reported  ICC or weighted Kappa < 0.70 |
| Measurement error | **+**  **?**  **–** | SDC or LoA < MIC5  MIC not defined SDC or LoA > MIC5 |
| Hypotheses testing for construct validity | **+** | The result is in accordance with the hypothesis7 |
|  | **?** | No hypothesis defined (by the review team) |
|  | **–** | The result is not in accordance with the hypothesis7 |
| Cross‐cultural validity\measurement invariance | **+** | No important differences found between group factors (such as age, gender, language) in multiple group factor analysis OR no important DIF for group factors (McFadden's R2 < 0.02) |
|  | **?** | No multiple group factor analysis OR DIF analysis performed |
|  | **–** | Important differences between group factors OR DIF was found |
| Criterion validity | **+** | Correlation with gold standard ≥ 0.70 OR AUC ≥ 0.70 |
|  | **?** | Not all information for '+' reported |
|  | **–** | Correlation with gold standard < 0.70 OR AUC < 0.70 |
| Responsiveness | **+** | The result is in accordance with the hypothesis7 OR AUC ≥ 0.70 |
|  | **?** | No hypothesis defined (by the review team) |
|  | **–** | The result is not in accordance with the hypothesis7 OR AUC <  0.70 |

Key: AUC = area under the curve, CFA = confirmatory factor analysis, CFI = comparative fit index, CTT

= classical test theory, DIF = differential item functioning, ICC = intraclass correlation coefficient, IRT = item response theory, LoA = limits of agreement, MIC = minimal important change, RMSEA: Root Mean Square Error of Approximation, SEM = Standard Error of Measurement, SDC= smallest detectable change, SRMR: Standardised Root Mean Residuals, TLI = Tucker‐Lewis index.
