# Supplementary material for: A conceptualization and psychometric evaluation of positive psychological outcome measures used in adolescents and young adults living with HIV: A mixed scoping and systematic review
Source: PLOS Glob Public Health. 2024 Aug 12;4(8):e0002255. doi: 10.1371/journal.pgph.0002255 (PMC11318935; doi:10.1371/journal.pgph.0002255)
Supplement: S6 Table — (DOCX) [file pgph.0002255.s006.docx]

## S6 Table: GRADE checklist- best evidence synthesis [21]

| **Quality level** | **Definition/criterion** |
| --- | --- |
| High | We are very confident that the true measurement property lies close to that of the estimate of the measurement property |
| Moderate | We are moderately confident in the measurement property estimate: the true measurement property is likely to be close to the estimate of the measurement property, but there is a possibility that it is substantially different |
| Low | Our confidence in the measurement property estimate is limited: the true measurement property may be substantially different from the estimate of the measurement property |
| Very low | We have very little confidence in the measurement property estimate: the true measurement property is likely to be substantially different from the estimate of the measurement property |

## S7 Figure: Study selection


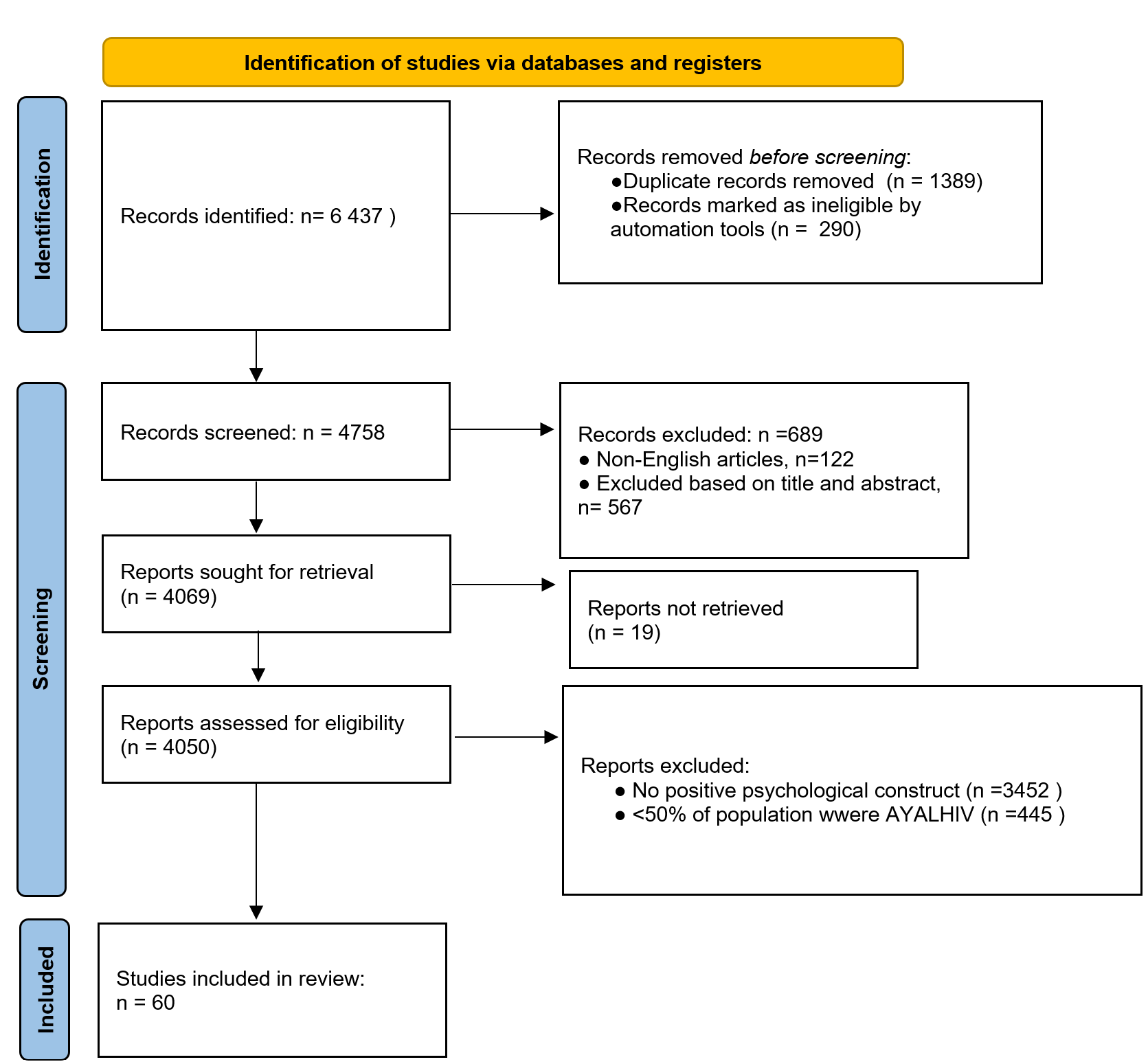


Online Supplementary File 6: Study selection

## S8 Table: Qualitative mapping of psychological constructs

| Construct | Theme |
| --- | --- |
| Family networks | **Social support** |
| Sense of belonging |  |
| Peer support |  |
| Social capital |  |
| Positive relations |  |
| Social network |  |
| Self-esteem | **Self-concept** |
| Self-reliance |  |
| Self-acceptance |  |
| Self-reflection |  |
| Treatment adherence | **Resilience** |
| Resilience |  |
| Coping |  |
| Social competence |  |
| Belief systems |  |
| Courage |  |
| Stigma | **Barriers to positive psychological constructs** |
| Fear of self-disclosure |  |

## S9 Table: Outcomes utility

| **Outcome** | **Country of origin; year** | **Classification** | **Population -condition/s** | **Completion Time** | **Response categories** | **N items** | **Score range** | **Criteria values** | **Scoring manual: meaning of higher score** | **Cost** |
| --- | --- | --- | --- | --- | --- | --- | --- | --- | --- | --- |
| Body appreciation scale 2 (BAS-2) | USA; 2014 | Generic |  |  | 5 | 10 | 5–50 |  |  | free |
| Psychological Adjustment to Illness Scale Self Report (PAIS-SR) | USA/ France; 1990 | Generic | Medical patients | 20-25 mins | 4 | 46 | 0–3 |  | poorer psychosocial adjustment | $30 |
| Acceptance of illness scale (AIS) | USA; 2016 | Generic | Adults |  | 5 | 8 | 8-40 |  | good disease acceptance | Free |
| Flourishing Well Being Scale (FBWS) | USA; 2009 | Generic | adults | 1 min | 7 | 8 | 8-56 | 50^th^ percentile – typica/ healthy) levels of flourishing  <15^th^ Percentile- low well-being. | person with many psychological resources and strengths. | Free |
| HIV meaningfulness scale (HIVMS) | USA; 2015 | Condition-specific | PLHIV | 5 mins | 7 | 4 | 4–28 |  | high level of meaningfulness | Free |
| Mastery Scale (MS) | USA; 1978 | Generic | Adults, 18+ | 5 mins | 4 | 7 | 7-28 |  | greater levels of mastery. | Free |
| Positive Outlook-Individual Protective Factors Index (IPFI) | USA; 1992 | Generic | Low-income students in grades 7-11. | 5 mins | 4 | 6 | 6-24 |  | high positive outlook. | Free |
| Child Youth Resilience Measure-12 (CYRM-12) | Multinational; 2009 | Generic | Children and Youth, 5 -23 years | < 15 mins | 5 | 12 | 12–60 |  | the more resilience components are present | Free |
| Connor-Davidson Resilience scale-25 (CDRS-25) | USA; 2003 | Generic | Adolescent to elderly adult | >5 mins | 5 | 25 | 0-100 |  | higher resilience | Not free Variable price |
| Connor-Davidson Resilience scale (CDRS-10) | USA; 2007 | Generic | Adolescent to elderly adult | 5 mins | 5 | 10 | 0–40 |  | higher resilience | Not free Variable price |
| Adolescent HIV Self-Management Scale | South Africa; 2018 | Condition-specific | ALHIV |  | 4 | 44 | 0-132 |  |  |  |
| Self-compassion scale (SCS) | USA; 2010 | Generic | Adults | 2 mins | 5 | 12 | 5–100 | average scores = 3.0  low self-compassion = 1-2.5  high self-compassion = 3.5-5.0 | high levels of self-compassion | Free |
| Beck Youth Self-Concept Scale (BYSCS) | USA; 2005 | Generic | Children 7–18 |  | 4 | 20 | 0–60 |  | higher distress | $270 |
| Tennessee Self-concept Scale-2 (TSCS-2) | USA; 1997 | Generic | children aged 7–14  Adults 13+ | 10-20 mins | 5 | 20 | 0-80 |  | higher self-concept and self-esteem |  |
| Self-Efficacy Questionnaire for Children (SEQC) | Netherlands; 2001 | Generic | Adolescents 14-18 |  | 5 | 24 | 5-120 |  | lower level of depression. | Free |
| Self-Efficacy for Managing Chronic Disease 6-Item Scale (SE-6-Xhosa) | USA; 2001 | Condition-specific | chronic conditions |  | 10 | 6 | 6–60 |  | higher self-efficacy. | Free |
| HIV Adherence Self-Efficacy Assessment Survey (HIV-ASES) | USA; 2007 | Condition-specific | HIV patients |  | 10 | 12 | 0-120 | <90 is cut-off for low self-efficacy | higher adherence self-efficacy |  |
| Rosenberg Self-esteem Measure (RSEM-10) | USA; 1965 | Generic | Adults and Adolescents |  | 4 | 10 | 4–40 | Normal range: 15-25  Low < 15 | greater self esteem | free |
| Modified Rosenberg Self-esteem Measure (RSEM-8) |  |  |  |  |  |  |  |  |  |  |
| Self-esteem-Hare Area-specific self-esteem scale (HASSES) | USA; 1975 | Generic | Youth 10-18 |  | 4 | 30 | 4–120 |  | higher self-esteem |  |
| Missoula Vitas Quality of Life Index (MVQOLI) Transcendence Subscale | USA; 1998 | Condition Specific | Palliative care Patients |  | 5 | 25 | 0-30 |  | higher QOL. | free |
| Ad hoc Self-efficacy to protect from unwanted sex |  | Generic |  |  | 4 | 4 | 4-16 |  | greater self-efficacy |  |
| Ad hoc Self-efficacy for correct condom use | South Africa; 2009 | Generic |  |  | 3 | 9 | 0–18 |  | greater self-efficacy |  |
| Ad hoc Self-efficacy for negotiating condom use | South Africa; 2009 | Generic |  |  | 3 | 4 | 0-12 |  | greater self-efficacy |  |
| Ad hoc Self-esteem | South Africa; 2009 | Generic |  |  | 4 | 6 | 0–24 |  | more positive self esteem |  |
| Ad hoc Self-esteem |  |  |  |  | 3 |  |  |  |  |  |
| Ad hoc Self–worth |  |  |  |  | 3 |  |  |  |  |  |
| Ad hoc Self-efficacy to disclose HIV |  |  |  |  |  | 5 |  |  |  |  |
